# Age-related cognitive task effects on gait characteristics: do different working memory components make a difference?

**DOI:** 10.1186/1743-0003-11-149

**Published:** 2014-10-27

**Authors:** Xingda Qu

**Affiliations:** Institute of Human Factors and Ergonomics, College of Mechatronics and Control Engineering, Shenzhen University, 3688 Nanhai Avenue, Shenzhen, Guangdong Province 518060 China

**Keywords:** Gait characteristics, Cognitive task, Working memory components, Spatial-temporal gait parameters

## Abstract

**Background:**

Though it is well recognized that gait characteristics are affected by concurrent cognitive tasks, how different working memory components contribute to dual task effects on gait is still unknown. The objective of the present study was to investigate dual-task effects on gait characteristics, specifically the application of cognitive tasks involving different working memory components. In addition, we also examined age-related differences in such dual-task effects.

**Methods:**

Three cognitive tasks (i.e. ‘Random Digit Generation’, ‘Brooks’ Spatial Memory’, and ‘Counting Backward’) involving different working memory components were examined. Twelve young (6 males and 6 females, 20 ~ 25 years old) and 12 older participants (6 males and 6 females, 60 ~ 72 years old) took part in two phases of experiments. In the first phase, each cognitive task was defined at three difficulty levels, and perceived difficulty was compared across tasks. The cognitive tasks perceived to be equally difficult were selected for the second phase. In the second phase, four testing conditions were defined, corresponding to a baseline and the three equally difficult cognitive tasks. Participants walked on a treadmill at their self-selected comfortable speed in each testing condition. Body kinematics were collected during treadmill walking, and gait characteristics were assessed using spatial-temporal gait parameters.

**Results:**

Application of the concurrent Brooks’ Spatial Memory task led to longer step times compared to the baseline condition. Larger step width variability was observed in both the Brooks’ Spatial Memory and Counting Backward dual-task conditions than in the baseline condition. In addition, cognitive task effects on step width variability differed between two age groups. In particular, the Brooks’ Spatial Memory task led to significantly larger step width variability only among older adults.

**Conclusion:**

These findings revealed that cognitive tasks involving the visuo-spatial sketchpad interfered with gait more severely in older versus young adults. Thus, dual-task training, in which a cognitive task involving the visuo-spatial sketchpad (e.g. the Brooks’ Spatial Memory task) is concurrently performed with walking, could be beneficial to mitigate impairments in gait among older adults.

**Electronic supplementary material:**

The online version of this article (doi:10.1186/1743-0003-11-149) contains supplementary material, which is available to authorized users.

## Introduction

Gait (walking) is one of the most common movement types in daily living. Gait is not carried out automatically, but demands cognitive resources[1] that are a key component in human information processing[2]. Dual-task paradigms have been used to determine the relative task demands of gait (considered as the primary task) and a concurrent secondary cognitive task. In dual-task conditions, gait characteristics have been quantified by various measures, such as local dynamic stability[3], margin of stability[4], and spatial-temporal parameters[5]. Dual-task gait changes have been found to be associated with cognitive function declines and increased risk of falls with aging[6].

Gait variability has also been examined in the dual-task conditions. Grabiner and Troy[7], for instance, reported that step width variability became smaller among young adults while performing the Stroop test during treadmill walking. They argued that such changes could be due to a voluntary adaptation of gait toward a more conservative gait pattern. By analyzing the same experimental data as in Grabiner and Troy[7], however, Dingwell et al.[8] suggested that decreased gait variability associated with the Stroop test cannot translate to greater gait stability.

In general, older adults are less cognitively capable compared to their younger counterparts[9], which may suggest that interference between cognitive tasks and gait are more severe in older adults. Age-related differences in gait have been reported in dual-task conditions. Springer et al.[10] found that swing time variability in older fallers increased when performing secondary cognitive tasks, but did not change in younger people. Older adults also tended to decrease walking velocity and increase stance time with the application of cognitive tasks, but younger adults showed little changes in these gait parameters[11].

Past research has examined the relationship between cognition and gait, and found that working memory that plays an essential role in human information processing was a significant predictor of gait velocity in a dual-task condition[12, 13]. There are three components in working memory: a modality-free central executive, a phonological loop, and a visuo-spatial sketchpad[14]. The central executive is responsible for the control and regulation of cognitive processes, such as planning, decision making, trouble shooting, etc. The visuo-spatial sketchpad is used primarily to store information in a visual or spatial code. The phonological loop maintains verbal information in a phonological or acoustic code. Note that the phonological loop can also be used to maintain visually presented verbal information (e.g., printed words) that is converted to a phonological code.

Researchers have reported that dual-task effects on postural control are different for the different aspects of working memory[15–17]. For example, it was found that maintaining a difficult posture interfered with visuo-spatial memory[15] and that age-related differences in postural control were significantly increased only when performing cognitive tasks involving the visuo-spatial memory[16]. A limitation with these studies is that postural control was only assessed using standing balance tasks. Though it is well recognized that gait characteristics are affected by concurrent cognitive tasks, and some researchers have even reported that dual-task-related gait changes were dependent on the types of cognitive tasks[6], how the different working memory components contribute to dual-task effects on gait is still unknown.

The objective of the present study was to investigate dual-task effects on gait characteristics with the application of cognitive tasks involving different working memory components. In addition, we examined age-related differences in such effects. Motor variability is an important feature of human movement[18], and gait variability has been widely studied and is considered to be of high clinical relevance[19–22]. Therefore, gait characteristics were assessed by spatial-temporal gait parameters and their variability. As discussed earlier, interference between standing postural control and cognitive tasks was found dependent on the working memory components involved in the cognitive tasks[15], and age-related differences in standing postural control differed among various working memory tasks[16]. Therefore, we hypothesized that dual-task effects on gait characteristics would differ between working memory components and between age groups.

To achieve the objective of this study, two phases of experiments were conducted. In the first phase, the perceived difficulty of a number of cognitive tasks was examined. Based on the results from the first phase, three cognitive tasks that were perceived to be equally difficult were identified and selected for the experiment in the second phase. In the second phase, four testing conditions were defined, corresponding to a baseline and the three equally difficult cognitive tasks. Gait data were collected under each testing condition, and the effects of various cognitive tasks on gait characteristics were determined.

## Methods

### Participants

Twelve young and 12 older participants from the local community took part in both phases (Table 1). There were six males and six females in each age group. The younger participants were between 20 to 25 years old, and the older participants were between 60 to 72 years old. To ensure that the participants of this study were cognitively capable of doing simple cognitive tasks, 50 one-digit and two-digit simple addition and subtraction problems were presented to potential participants at the beginning of Phase 1. Only those who were able to solve over 90% of these problems were included. In addition, all participants self-reported having no injuries, illness, or musculoskeletal disorders that could affect their normal gait patterns. Written informed consent, which was approved by the NTU Institutional Review Board, was obtained from each participant.

**Table 1:** **Demographic information of the participants**

	Young participants		Older participants	
	Mean	S.D.	Mean	S.D.
Age (year)	23.2	1.6	66.5	3.7
Body weight (kg)	56.8	7.4	60.3	7.1
Height (m)	169.5	10.4	160.2	7.9

### Phase 1: Assessment of the perceived difficulty of cognitive tasks

#### Procedure

Three types of cognitive tasks were examined including Random Digit Generation, Brooks’ Spatial Memory, and Counting Backward. These cognitive tasks involved different working memory components[16]. In particular, the working memory components primarily demanded by Random Digit Generation, Brooks’ Spatial Memory, and Counting Backward were the central executive, visuo-spatial sketchpad, and phonological loop, respectively[16].

Each cognitive task was defined at three difficulty levels. In the Random Digit Generation task, the participants were required to generate single digits (0 – 9) as randomly as possible, and to do so in time with a metronome beating at different rates. In particular, the low, medium and high difficulty levels of the Random Digit Generation task corresponded to rates of 30, 60, and 120 per minute, respectively. When performing the Brooks’ Spatial Memory task, a four by four grid board was placed in front of the participants. The experimenter read out a list of instructions for placing consecutive numbers in the grid at a standard rate of 3 seconds per instruction. The starting square was the second row of the second column of the grid. After the last instruction, the participants were asked to repeat back the list of instructions. The low, medium and high difficulty levels of the Brooks’ Spatial Memory task corresponded to four, five, and six instructions in a list, respectively. In the Counting Backward task, the participants were required to count aloud backward, in steps of 1 (i.e. low level of difficulty), 3 (i.e. medium level of difficulty), and 7 (i.e. high level of difficulty), respectively, as fast and accurately as possible, from a random number between 800 and 999 provided by the experimenter.

In the experiment, the participants were seated and practiced the selected cognitive tasks at each of the different difficulty levels. The three cognitive tasks (i.e., Random Digit Generation, Brooks’ Spatial Memory, and Counting Backward) were presented to the participants in a random order. There was at least a five-minute interval between the presentations of two consecutive cognitive tasks. When practicing each cognitive task, the three difficulty levels were presented to the participants in a random order as well. Practice of each cognitive task was stopped when the participants self-reported they were confident with the task. Right after practicing each cognitive task, the perceived difficulty of the task at different difficulty levels was assessed using a seven-point Likert scale as below:

#### Analysis and results

Comparisons of perceived difficulty among the three cognitive tasks were conducted using the Friedman two-way ANOVA. Since each difficulty level of a cognitive task was compared with each level of both other cognitive tasks, there were 27 combinations of the three cognitive tasks for comparison (3 levels of Random Digit Generation × 3 levels of Brooks’ Spatial Memory × 3 levels of Counting Backward). If the cognitive tasks with equal perceived difficulty were identified from the Friedman two-way ANOVA, the perceived difficulty of these cognitive tasks was also compared between the two age groups using Kolmogorov-Smirnov tests. The level of significance (α) was set at 0.05.

Table 2 summarizes the perceived difficulty ratings for the cognitive tasks. Results from the Friedman two-way ANOVA indicated that the perceived difficulty was not significantly different between Random Digit Generation at the high difficulty level, Brooks’ Spatial Memory at the medium difficulty level, and Counting Backward at the medium difficulty level (*p* = 0.347). Therefore, these three condition were selected for the experimental study in Phase 2. Results from the Kolmogorov-Smirnov tests revealed that there was no significant difference between the two age groups in the perceived difficulty of Random Digit Generation at the high difficulty level (*p* = 0.100) and Brooks’ Spatial Memory at the medium difficulty level (*p* = 0.847). However, older adults perceived Counting Backward at the medium difficulty level to be more difficult than did younger adults (*p* = 0.010).

**Table 2:** **Perceived difficulty ratings for cognitive tasks: Mean (SD)**

Difficulty level	Cognitive tasks					
	Random Digit Generation		Brooks’ Spatial Memory		Counting Backward	
	Young	Old	Young	Old	Young	Old
Low	1.42 (0.67)	2.83 (0.83)	3.17 (0.94)	3.33 (1.15)	1.33 (0.49)	3.25 (0.97)
Medium	2.00 (0.85)	3.75 (0.75)	4.17 (0.72)	4.67 (1.07)	3.17 (0.94)	5.00 (0.95)
High	3.42 (1.24)	4.83 (1.11)	5.75 (0.45)	6.00 (0.95)	4.92 (1.00)	6.42 (0.79)

### Phase 2: Effects of various cognitive tasks on gait measures

#### Experimental procedure

**Figure 1:**
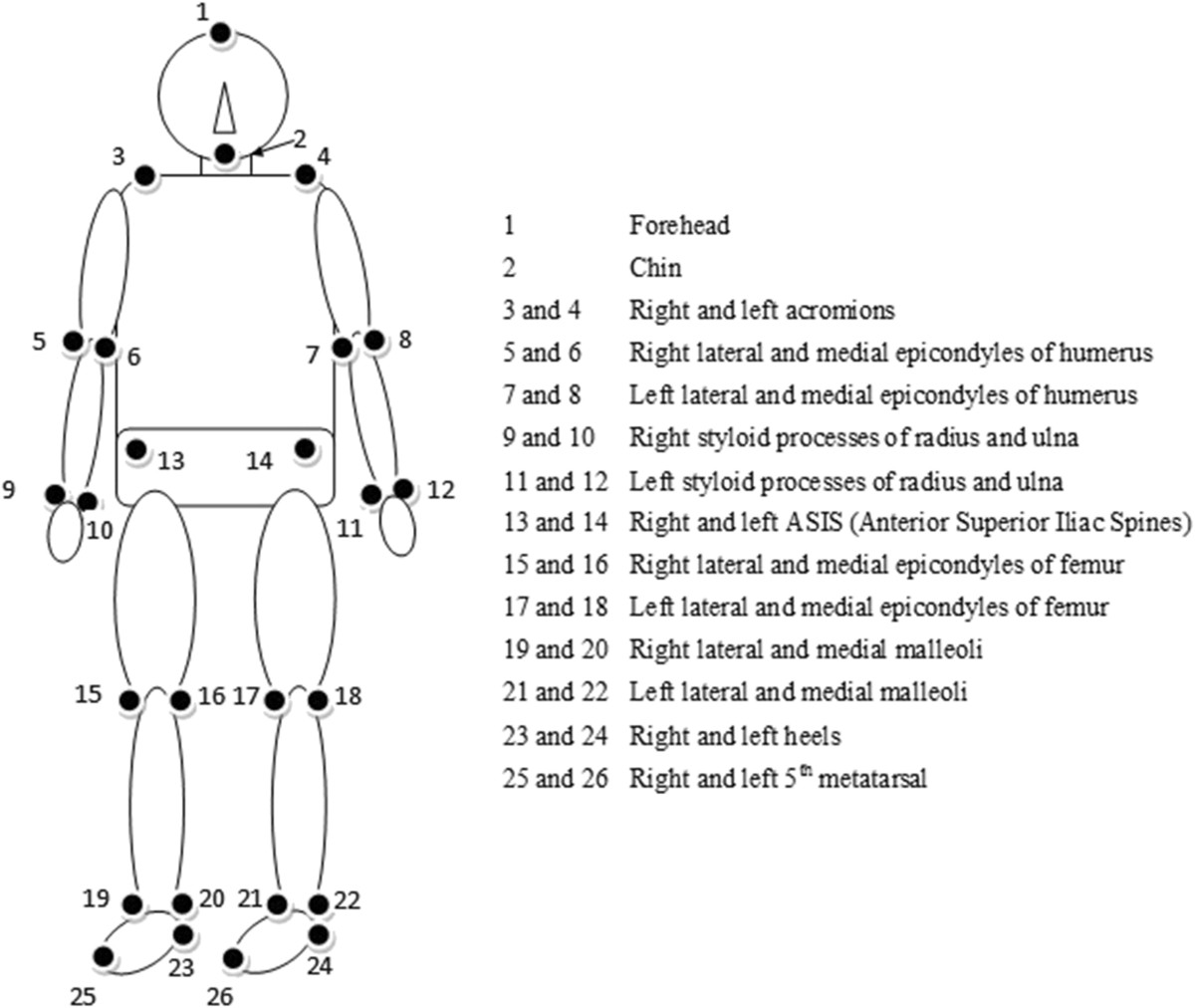
**Marker placement on the human body.**

The second phase was started one month after completing Phase 1, and the same group of participants as in Phase 1 were involved. At the beginning of the experiment, participants changed into tight-fitting suits, and 26 reflective markers were placed bilaterally over selected anatomical landmarks of the body (Figure 1). This marker placement scheme was used to track several body segments, including the head, trunk, upper arms, lower arms, thighs, shanks, and feet. A test was then conducted to determine the participants’ comfortable walking speed. In this test, the participant was asked to walk on a treadmill (Biodex RTM 600, Shirley, NY, USA) at a relatively low initial speed. Then, the treadmill speed was increased by a small amount (i.e. 0.1 mph) on each successive trial until the participants reported that they felt uncomfortable with the speed. The treadmill speed was then increased further and slowly decreased by the same small amount on each successive trial until the speed was reported to be comfortable. The average of the transition speeds was taken as the comfortable treadmill speed. Note that each participant was tested at their comfortable treadmill speed for all trials. There was a significant difference between the two age groups in treadmill speed (Young: 0.73 ± 0.12 meters/second; Old: 0.54/±0.26 meters/second; *p* = 0.028).

Subsequently, participants were given a 10-minute practice period in which they performed treadmill walking and cognitive tasks concurrently. After this practice, body kinematic data were collected using an eight-camera motion capture system (Motion Analysis Eagle System, CA, USA) at a sampling rate of 100Hz while participants walked naturally on the treadmill at their self-selected comfortable speed wearing standard shoes. There were four testing conditions, corresponding to a baseline and the three selected cognitive tasks (i.e. Random Digit Generation at high difficulty level, Brooks’ Spatial Memory at medium difficulty level, and Counting Backward at medium difficulty level). One four-minute treadmill walking trial was completed in each testing condition. In the baseline condition, no cognitive task was performed by participants during walking. In the cognitive task conditions, participants were instructed to prioritize the treadmill walking task (primary task) over the cognitive task (secondary task), with the latter initiated about 10 seconds after the start of walking and continued until the end of the walking trials. Participants’ responses to the cognitive tasks were audio recorded. Data in the initial 20 seconds and last 10 seconds in each trial were removed to avoid initial transients and termination anticipation effects, respectively. The interval between two consecutive walking trials was at least two minutes to minimize carry-over effects and possible confounding effects caused by fatigue. In addition, in order to minimize order effects, the testing conditions (including the baseline) were presented to participants in a random order.

#### Gait measures

Raw data from the motion capture system were filtered using a second order, low pass Butterworth filter with a cut-off frequency of 6 Hz. One hundred and fifty steps (i.e. 75 strides) were then randomly selected from each trial for the calculation of gait measures. Spatial-temporal gait parameters were determined, including step length, step width and step time. Step length and step width were measured as the anterior-posterior distance and medial-lateral distance between sequential left and right heel-strikes, respectively. Step time was defined by the time interval between sequential left and right heel-strikes. Heel strikes were determined when the heel markers in the vertical direction showed a local minimum within a gait cycle.

The standard deviations of these gait parameters were first calculated from left and right steps separately, and then combined using the equation suggested by Galna et al.[23].

1 $$SD_{\mathrm{Combined}}=\sqrt{\frac{SD_{\mathrm{Left}}^{2}+SD_{\mathrm{Right}}^{2}}{2}}$$

where *SD*_*Combined*_, *SD*_*Left*_, and *SD*_*Right*_ represent the combined standard deviation, standard deviation from left steps, and standard deviation from right steps, respectively. The combined standard deviations were used to account for gait variability. Besides these measures of gait variability, mean values of step length, step width and step time were calculated as well.

#### Measures of cognitive task performance

Measures of cognitive task performance were derived from what were used in Maylor and Wing[16]. In particular, the performance during the Random Digit Generation task was measured using redundancy, which is defined based on the frequency of successive pairs of digits. Details on the redundancy calculation were provided in[24]. Performance in the Brooks’ Spatial Memory task was the percentage of correct responses, which was defined by the ratio between the number of correct responses and the total number of responses. A response in the Brooks’ Spatial Memory task was considered to be correct only when all the instructions in the list were repeated back correctly. The performance measure during the Counting Backward task was the percentage of correct subtractions, defined by the ratio between the number of correct subtractions and the total number of subtractions.

#### Analysis

Two-way analysis of variance (ANOVA) was carried out on the gait measures with ‘age’ and ‘cognitive task’ as the independent variables. ‘Age’ was a between-subject factor defined at two levels: old versus young. ‘Cognitive task’ was a within-subject factor that had four levels corresponding to the baseline (no cognitive task), Random Digit Generation task, Brooks’ Spatial Memory task, and Counting Backward task, respectively. In case of a significant interaction between ‘age’ and ‘cognitive task’, further statistical tests were conducted on the gait measures where interaction was found. In particular, one-way ANOVA was conducted for each age group with ‘cognitive task’ as the independent variable. Post hoc pairwise comparisons were conducted using the Bonferroni correction when necessary. In addition, cognitive task performance was compared between the two age groups using two-sample *t*-tests. Significance was concluded when *p* <0.05.

## Results

Cognitive task significantly affected step time and step width variability (Table 3). Post hoc pairwise comparisons showed that the Brooks’ Spatial Memory task led to longer step times (*p* = 0.009) compared to the baseline condition, and step width variability was smaller in the baseline condition compared to the Brooks’ Spatial Memory (*p* = 0.017) and Counting Backward dual-task conditions (*p* = 0.020). Significant age effects were found in step time variability, step time, step length, and step width (Table 3). In particular, older participants had larger step time variability, longer step times, smaller step lengths, and larger step widths.

**Table 3:** **Results from ANOVA: Mean (SE)**

		Age				Cognitive tasks						Age × Cognitive tasks	
		Young	Old	***F*** ^(1, 88)^	***p***	Baseline	Random Digit Generation	Brooks’ Spatial Memory	Counting Backward	***F*** ^(3, 88)^	***p***	***F*** ^(3, 88)^	***p***
Step time (ms)	*Variability*	29.8 (2.1)	50.5 (3.7)	22.820	<0.001*	45.5 (6.4)	38.8 (4.0)	38.7 (4.2)	37.7 (4.1)	0.678	0.568	0.109	0.955
	*Mean*	627.3 (10.6)	673.9 (17.0)	5.815	0.018*	597.0 (20.6)	659.0 (22.5)	683.8 (17.2)	662.6 (18.0)	3.744	0.014*	0.586	0.626
Step length (mm)	*Variability*	47.1 (6.5)	47.0 (5.9)	<0.001	0.991	45.4 (7.7)	44.0 (8.2)	53.3 (10.6)	45.5 (8.4)	0.223	0.880	0.447	0.720
	*Mean*	542.2 (17.7)	419.1 (29.9)	12.362	0.001*	458.6 (35.1)	444.1 (31.4)	520.9 (46.4)	498.9 (32.6)	1.027	0.385	0.534	0.660
Step width (mm)	*Variability*	17.3 (0.7)	18.3 (0.8)	1.117	0.293	15.1 (0.5)	17.7 (1.1)	19.1 (1.0)	19.2 (1.1)	4.115	0.009*	2.855	0.042*
	*Mean*	79.4 (3.9)	108.7 (8.4)	10.502	0.002*	77.3 (7.9)	92.4 (9.6)	108.3 (10.9)	98.0 (9.6)	2.056	0.112	1.374	0.256

*indicates statistical significance.

**Figure 2:**
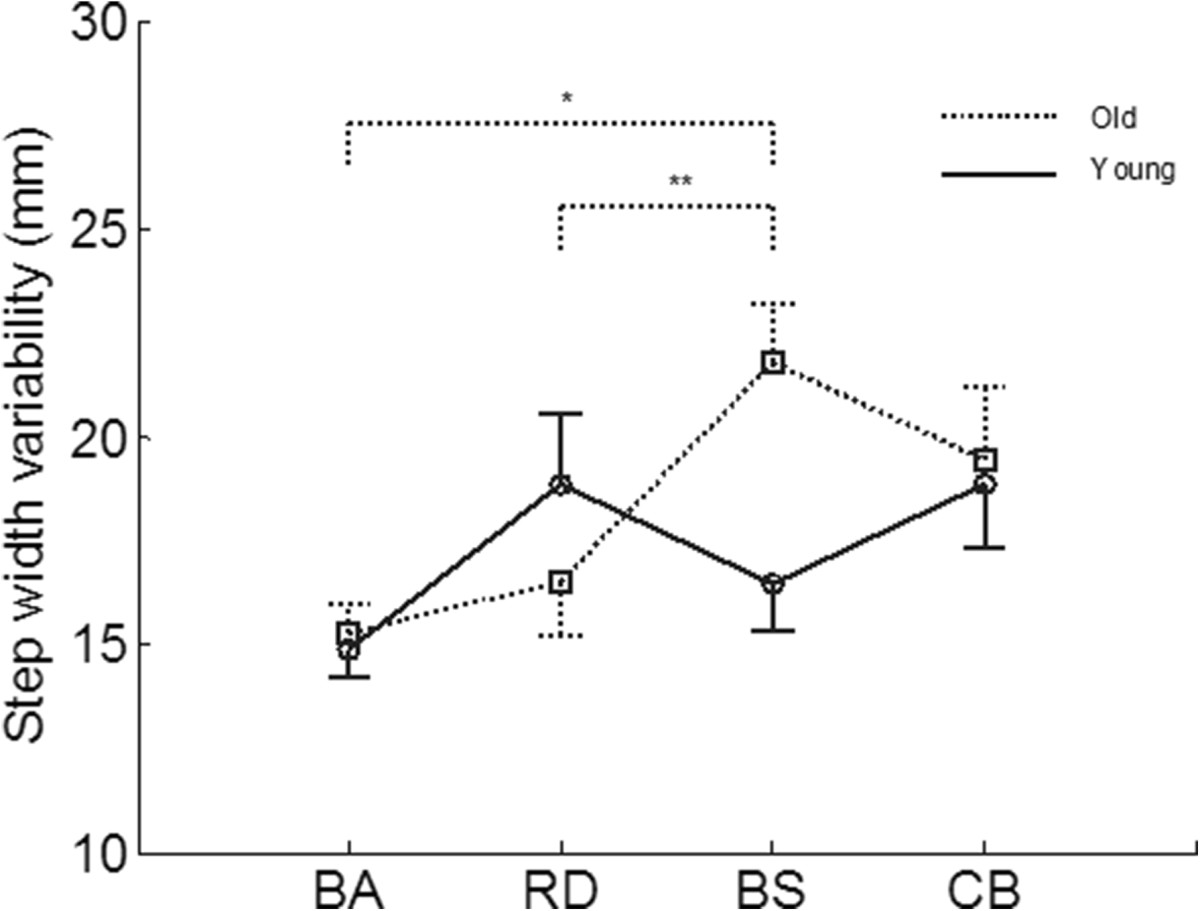
**Interaction between age and cognitive task on step width variability.** *p <0.05; **p < 0.10. BA = Baseline; RD = Random Digit Generation; BS = Brooks’ Spatial Memory; CB = Counting Backward.

Significant interaction effects between age and cognitive task were found in step width variability (Table 3). In the older group, step width variability in the Brooks’ Spatial Memory dual-task condition was larger than that in the baseline condition (*p* = 0.009). In addition, the difference in step width variability between the Brooks’ Spatial Memory and Random Digit Generation dual-task conditions approached significance in the older adults (*p* = 0.053) (Figure 2). However, the step width variability of young participants was not affected by the cognitive tasks (Figure 2).

Under the dual-task conditions, older participants performed worse in the Brooks’ Spatial Memory and Counting Backward tasks. But, no difference in the performance of the Random Digit Generation task was found between the two age groups in the dual-task conditions (Table 4).

**Table 4:** **Cognitive task performance measures under dual-task conditions: Mean (SE)**

	Young	Old	***p***
Random Digit Generation: % Redundancy	18.91 (1.24)	19.48 (1.50)	0.770
Brooks’ Spatial Memory: % Correct Response	97.22 (1.87)	63.89 (8.42)	<0.001*
Counting Backward: % Correct Subtraction	97.08 (0.48)	94.83 (0.94)	0.045*

*indicates statistical significance.

## Discussion

The main objective of this study was to determine how different working memory components contribute to gait characteristics in dual-task conditions. Three cognitive tasks (i.e. Random Digit Generation, Brooks’ Spatial Memory, and Counting Backward) were examined that involve different working memory components. In order to make these three cognitive tasks comparable with each other, each of them was defined at three difficulty levels and their perceived difficulty was assessed in the first phase of the study. In the second phase, gait characteristics quantified by spatial-temporal gait parameters were measured under a baseline (i.e. a no cognitive task condition) and three equally difficult cognitive task conditions.

We found that cognitive tasks involving different working memory components had distinct effects on step width variability. In particular, step width variability increased with the concurrent Brooks’ Spatial Memory and Counting Backward tasks. Step width is related to medial-lateral postural control during locomotion, while variability is linked to postural control error and noise[25]. Therefore, larger step width variability in the Brooks’ Spatial Memory and Counting Backward dual-task conditions indicates that these tasks led to worse medial-lateral postural control during locomotion. This finding seems inconsistent with Grabiner and Troy[7], who reported that step width variability decreased among young adults with the application of a concurrent cognitive task. This discrepancy is likely due to two reasons. First, the selected cognitive task in Grabiner and Troy[7] was the Stoop test, which demands cognitive resources from both the central executive and visuo-spatial sketchpad and is different from the cognitive tasks examined in the present study in terms of difficulty levels and involved working memory components. Second, only younger participants were recruited in Grabiner and Troy[7], while both younger and older adults participated in our study. We did not found any differences in step width variability under different testing conditions among younger people either (Figure 2). Compared to their older counterparts, younger people have sufficient cognitive resources to perform the cognitive task and gait concurrently, which helps avoid any decrement in postural control performance during gait.

The application of the concurrent Brooks’ Spatial Memory task also led to longer step times than in the baseline condition. Longer step time is an attempt to improve postural stability by reducing the center-of-mass forward momentum (i.e., the anterior velocity)[26]. Therefore, this finding suggests that people are able to adaptively adjust their gait patterns to minimize adverse effects of the Brooks’ Spatial Memory task’s on their postural stability in the dual-task condition.

In a dual-task condition, there might be between-task interference if the two tasks share the same information processing pathways[2]. Visual input plays an important role in postural control by providing the neural controller with continuous updated information regarding body orientation and movements[27]. Therefore, postural control during gait requires the visual information processing pathway. The working memory component demanded by the Brooks’ Spatial Memory task is the visuo-spatial sketchpad. In other words, the Brooks’ Spatial Memory task and postural control task during gait share the same information processing pathway. This can help explain why step time and step width variability was affected by the concurrent Brooks’ Spatial Memory task.

The concurrent Counting Backward task was also associated with larger step width variability. This finding suggests that medial-lateral postural control during locomotion also demands cognitive resources from the phonological loop. Further, both the Counting Backward task and gait are rhythmic tasks. It was reported that interference existed between two concurrent rhythmic tasks with different frequencies[28]. Compared to the visuo-spatial sketchpad and phonological store, medial-lateral postural control during gait appears to place less demands on the central executive component, since no significant effects on step width variability were found in the Random Digit Generation dual-task condition. Though the three cognitive tasks (i.e. Random Digit Generation, Brooks’ Spatial Memory, and Counting Backward) affected step width variability differently, an interesting finding is that no significant difference was found between them. A possible explanation for this is that the sample was too small (n = 12 in each age group) to make the difference between them statistically significant.

Step time variability and step length variability were not affected by the selected concurrent cognitive tasks. One explanation for this finding is that step time variability and step length variability were less sensitive descriptors of dual-task-related changes in postural control during locomotion compared to step width variability. Step length is related to anterior-posterior postural control during locomotion. Thus, this may suggest that dual-task interference with anterior-posterior postural control was less severe than that with medial-lateral postural control. Another explanation for this finding is that gait was performed on a treadmill, which reduces variability in the anterior-posterior direction because of the direction of the treadmill motion. Medial-lateral variability would therefore be less affected by treadmill walking.

We also examined age-related differences in gait characteristics. We observed that step time variability was larger in older adults. Increased step time variability is associated with an increased risk of future falls[19]. Thus, this finding supports that fall risk is higher in older adults. This finding also suggests that step time variability could be a sensitive descriptor of locomotion control of older and younger people.

Older adults had longer step times and shorter step lengths. This finding is consistent with a previous investigation[29] and indicates that older adults adopt a more cautious gait pattern to stabilize their postures in the dual-task condition[25]. Older adults also showed larger step widths. To maintain postural balance, the vertical projection of the center of mass should be within the base of support (BOS). Larger step width is associated with larger BOS in the medial-lateral direction. Previous studies suggested that control of medial-lateral stability was a major problem associated with increased risk of falls in older adults[30]. Thus, adopting larger step width is a postural control strategy for older adults to improve their medial-lateral postural stability.Cognitive task effects on step width variability were different between the two age groups (Figure 2). In particular, the concurrent Brooks’ Spatial Memory task led to significantly larger step width variability only in older adults. In the Brooks’ Spatial Memory dual-task condition, the primary task is treadmill walking and the Brooks’ Spatial Memory task is the secondary task. Therefore, this finding indicates that the secondary Brooks’ Spatial Memory task had greater interference with the primary treadmill walking in older adults. Such greater interference may have resulted from age-related decreases in visual-spatial information processing capability, which led to less cognitive resources from the visual-spatial sketchpad allocated to the primary treadmill walking task in older adults versus younger adults.

No age-related differences were found when performing the concurrent cognitive tasks other than the Brooks’ Spatial Memory task. Thus, we may conclude that compared to the other working memory components, age-related decrements in information processing capability with the visuo-spatial sketchpad become more severe. This finding is consistent with Maylor and Wing[16], who reported that age-related differences in postural stability significantly increased only when performing cognitive tasks involving the visuo-spatial sketchpad component of working memory.

One limitation of the present study is that the perceived difficulty ratings for cognitive tasks were subjective. Older adults may have considered themselves as capable as their younger counterparts. If so, they might rate the cognitive tasks as less difficult than what they actually perceived. This may help explain why the Brooks’ Spatial Memory task performance was worse in older adults under the dual-task condition (Phase 2), while there was no difference in the perceived difficulty of the Brooks’ Spatial Memory task between younger and older adults in the single-task condition (Phase 1). It was also found that older adults perceived Counting Backward to be more difficult than did younger adults under the single-task condition. At the same time, older participants performed worse in the Counting Backward tasks under the dual-task conditions. Thus, the perception of the Counting Backward task difficulty could be a predictor of the Counting Backward task performance in the dual-task condition.

Another limitation is that gait data were collected during treadmill walking. Due to the low demands on physical lab space, treadmills have been widely used in gait analysis. However, the data collected during treadmill walking may not completely reflect the overground gait patterns that are more common in daily living[31]. Also, we did not assess cognitive task performance in single task conditions in our experiment, so estimation of dual-task costs becomes impossible.

## Conclusion

The major contribution of the present study is that it is the first attempt to provide knowledge about how different working memory components contribute to dual task effects on gait characteristics and the age-related differences in such effects. Such knowledge can serve as a basis for the development of fall prevention interventions for older adults. In particular, cognitive tasks involving the visuo-spatial sketchpad interfered with gait more severely in older adults versus younger adults. Previous research showed that dual-task training can improve postural control performance[32]. Therefore, dual-task training in which a cognitive task involving the visuo-spatial sketchpad (e.g. the Brooks’ Spatial Memory task) is concurrently performed with walking could be beneficial to mitigate impairments in gait in older adults. A study investigating the effects of such dual-task training on fall risks in older adults should be conducted in future research.

## Authors’ original submitted files for images

Below are the links to the authors’ original submitted files for images. Authors’ original file for figure 1 Authors’ original file for figure 2
