# Supplementary figures and images for: Age-related cognitive task effects on gait characteristics: do different working memory components make a difference?
Source: J Neuroeng Rehabil. 2014 Oct 27;11:149. doi: 10.1186/1743-0003-11-149 (PMC4221663; doi:10.1186/1743-0003-11-149)

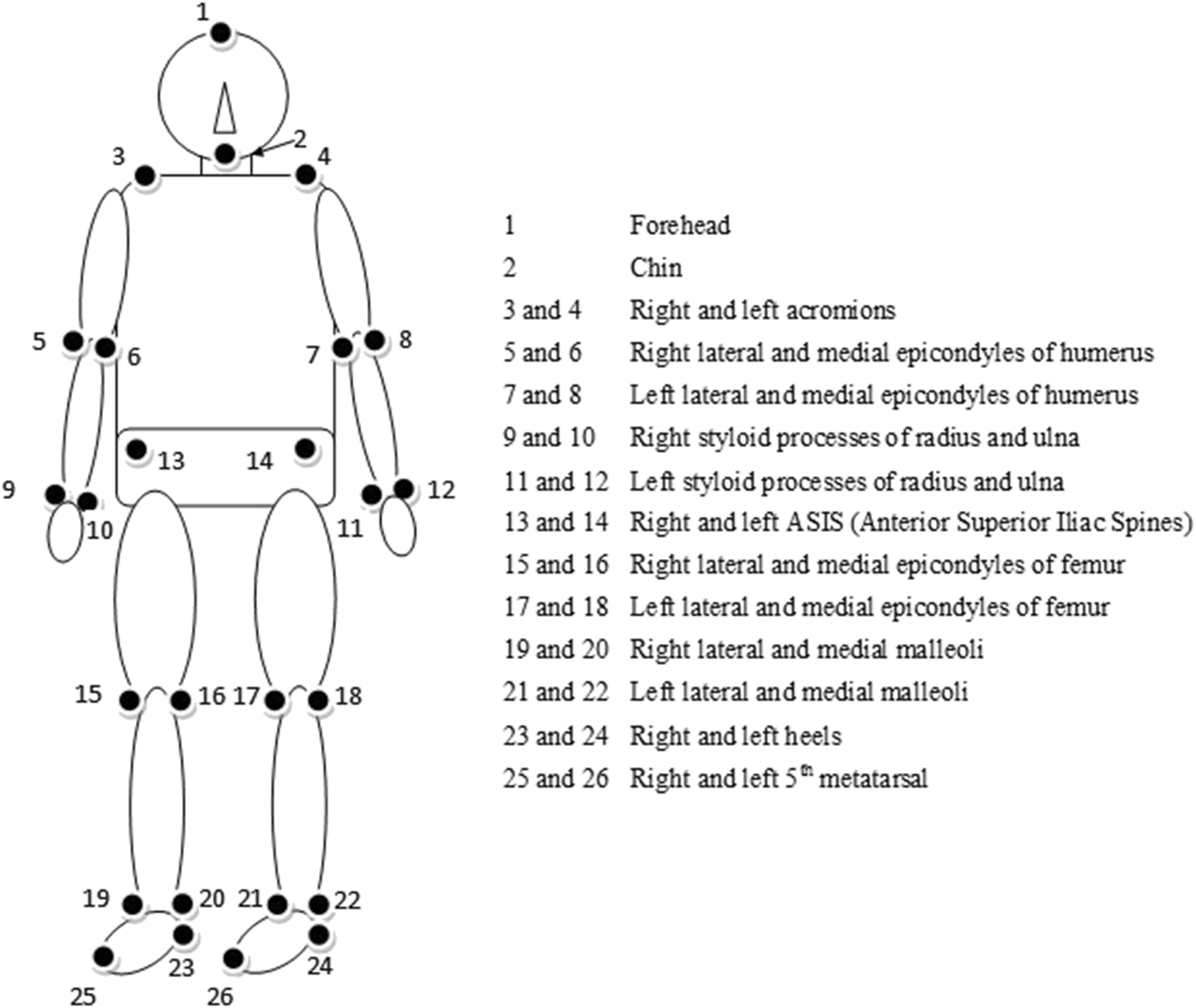

Supplement: Supplementary file 1 — Authors’ original file for figure 1 [file 12984_2014_666_MOESM1_ESM.tif]

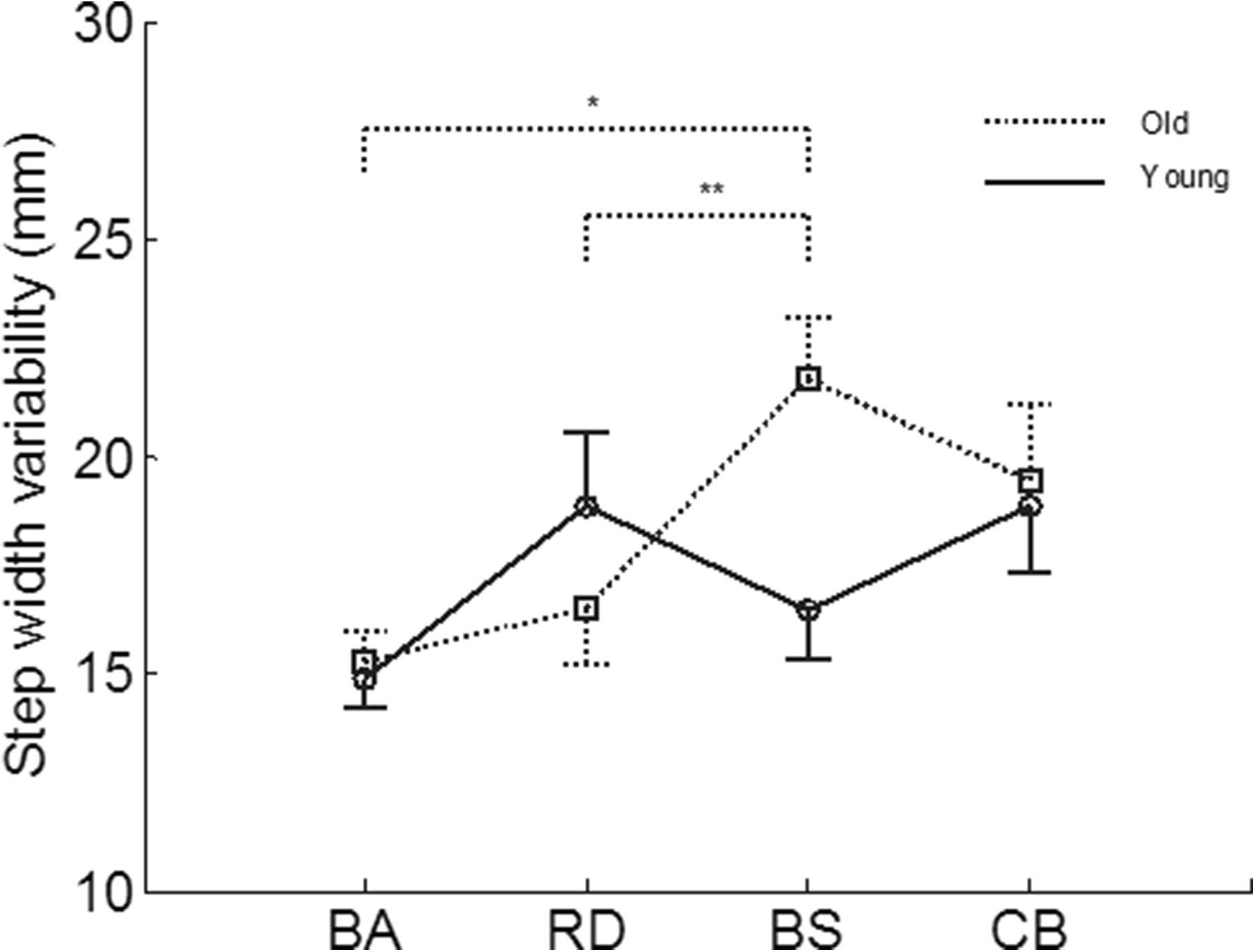

Supplement: Supplementary file 2 — Authors’ original file for figure 2 [file 12984_2014_666_MOESM2_ESM.tif]
